# An Early Functional Unsupervised Rehabilitation Protocol Allows Safe Return to Function After Achilles Tendon Repair

**DOI:** 10.7759/cureus.52477

**Published:** 2024-01-18

**Authors:** Leonidas Mitrogiannis, George Mitrogiannis, Kalliopi Melaki, Athanasios Karamitros, Apostolos Karantanas, Odysseas Paxinos

**Affiliations:** 1 Orthopedics Department, 251 Hellenic Air Force General Hospital, Athens, GRC; 2 Radiology Department, General Hospital of Piraeus "Tzaneio", Athens, GRC; 3 Radiology Department, University of Crete Medical School, Heraklion, GRC

**Keywords:** ultrasound, surgery, early functional, rehabilitation, achilles tendon

## Abstract

Purpose: Acute Achilles tendon tears in young active individuals are often treated surgically with prolonged rehabilitation, with the leg initially immobilized in plantar flexion in serial non-weight bearing casts and gradually progressing to full weight bearing. This study aimed to evaluate the safety of an early functional unsupervised rehabilitation protocol.

Methods: The medical records of 25 patients treated with open repair were available for follow-up. In 10 patients, an early functional unsupervised rehabilitation protocol was used with a removable cast, active range of motion exercises of the ankle from the first postoperative day, and full weight bearing in a walking boot with the foot plantigrade after the second week. Another 15 patients who followed the classic rehabilitation protocol were used as controls. The patients were evaluated with the Victorian Institute of Sports Assessment-Achilles (VISA-A) and an ultrasound examination-based score. A Wilcoxon test was used to compare the scores between the groups.

Results: The mean VISA-A score was 90.1 (SD = 9.87) for the early functional rehabilitation protocol group, while it was 83.8 (SD = 17.06, p = 0.624) for the control group. The mean ultrasound score was 7.75 (SD = 1.71) for the early functional rehabilitation protocol group, while it was 7.60 (SD = 3.05, p = 0.414) for the control group. There were no intra- or early postoperative complications in the groups, and all patients were satisfied with the results of their operation.

Conclusions: An early unsupervised functional rehabilitation protocol after open Achilles repair may allow for safe early mobilization and minimize the need for physiotherapy. The small number of participants is a limitation of this study, and further evaluation with more patients is necessary to document the efficacy.

## Introduction

The Achilles tendon is one of the largest tendons in the human body, resisting during sports loads up to 10-12.5 times body weight [1,2]. Achilles ruptures account for approximately 20% of large tendon injuries [3], with male dominance [4] and two age peaks: one under 40 and one over 60 years of age [5,6].

The most common mechanism of injury is a forceful eccentric contraction of the gastrocnemius and soleus muscles during sports [7]. Predisposing factors include chronic use of steroids, fluoroquinolones, and tendinopathy [8,9].

Most of the time, the diagnosis is clinical [10]. Magnetic resonance imaging (MRI) helps locate the extent of the injury; however, it is costly and not readily available [11]. Ultrasonography allows for excellent imaging of these injuries and is cheaper and widespread [12].

There is still a debate in the literature on the recommended treatment, with several surgical options and conservative methods available [10,13]. Open, mini-open, and percutaneous techniques [14-18] as well as augmented and non-augmented techniques [19] have been described. Compared to conservative treatment, surgery leads to better outcomes in terms of re-rupture rates, return to sports, calf atrophy, and range of motion [20,21]. On the other hand, the risk of infection and neuromuscular injury is higher in the surgically treated patients.

The classic rehabilitation protocol following surgical repair consists of 6-12 weeks of immobilization in plantar flexion with a non-weight bearing cast, which gradually progresses to full weight bearing and a plantigrade foot [21].

The purpose of this level-III retrospective study was to evaluate an early functional unsupervised rehabilitation protocol that allows the patient to walk full weight bearing on a walking boot with the foot plantigrade after the second postoperative week.

## Materials and methods

The medical records of 52 patients who were treated for acute Achilles tendon tear between 2013 and 2018 were retrieved from the hospital's database. A complete follow-up evaluation was available for 37 of the 52 patients. Twelve of these patients have been treated conservatively and were excluded, leaving 25 operatively treated patients to be included in this study. All patients had an acute, isolated, primary rupture that was treated with an open end-to-end repair.

Of these 25 patients, 10 had followed the early functional unsupervised rehabilitation protocol (Group A), while 15 patients followed the classic rehabilitation protocol under physiotherapy supervision (Group B, control group). Follow-up consisted of clinical evaluation, a simple visual pain score, and a rating of subjective satisfaction with the operative result. Posttraumatic Achilles tendinopathy was evaluated using the mean Victorian Institute of Sports Assessment-Achilles (VISA-A) score [22,23], while ultrasonography was used to assess the postoperative quality and tendinopathy of the tendon. The research was approved by the research and ethics committee of the hospital.

Surgical technique

**Figure 1:**
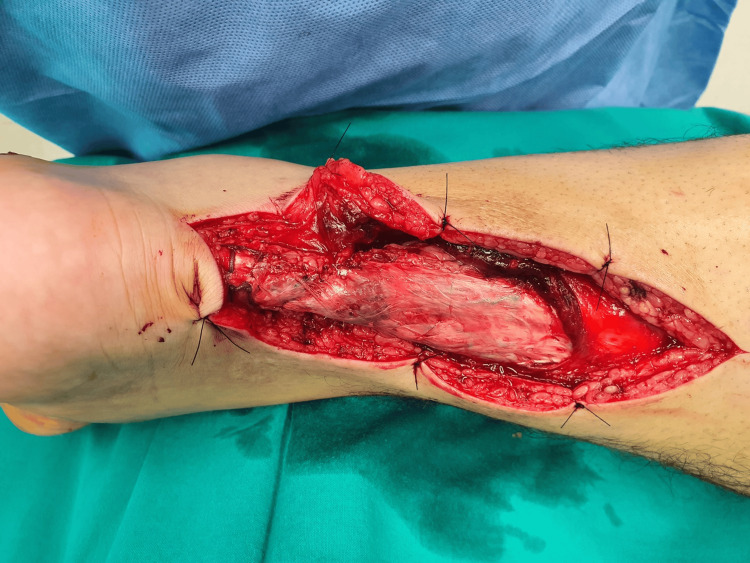
Intraoperative picture Full-thickness skin and paratenon flaps were meticulously elevated. The Achilles tendon was repaired with No. 1 PDS absorbable Krakow sutures. The palmaris was used as a cover flap over the repair site. PDS: Polydioxanone.

With the patient prone on the surgical table, under general or spinal anesthesia, an S-type skin incision was performed with the transverse leg just above the tear and the distal part just lateral to the Achilles, to protect the sural nerve. Full-thickness flaps including skin and para-tendon were carefully elevated, and the two stumps were approximated with Krakow sutures, using absorbable No. 1 polydioxanone (PDS) sutures. All patients had a plantaris tendon which was fashioned as a sheet of fascia and sutured over the repair site (Figure 1).

At the end of the surgery, the strength of the repair was clinically evaluated by stretching the foot in a plantigrade position. In all cases, the repair site was firmly approximated even under full tension.

Rehabilitation protocol

On the first postoperative day, patients of Group A were instructed by the surgeon to remove the splint and perform active range of motion exercises of the ankle as tolerated, twice daily. After two weeks, all dressings were removed, and the patients were allowed to walk full weight bearing with the foot plantigrade in a walking boot. At six weeks postoperatively, the boot was discarded, and the patients were allowed to walk with normal shoes using a wedged 1-cm height insole for another six weeks. Full return to unrestricted activities was allowed four months after the operation. Rehabilitation was unsupervised in all patients of Group A. All patients felt confident to perform their exercises unsupervised after brief instruction on the first postoperative day before they were discharged.

The patients in the control group (Group B) followed the classic rehabilitation protocol, with the leg initially immobilized in plantar flexion (20°) in serial non-weight bearing casts and then gradually progressing to full weight bearing with a plantigrade foot during a period of 6-12 weeks postoperatively. A physiotherapist supervised the rehabilitation of Group B patients.

Ultrasound examination

**Figure 2:**
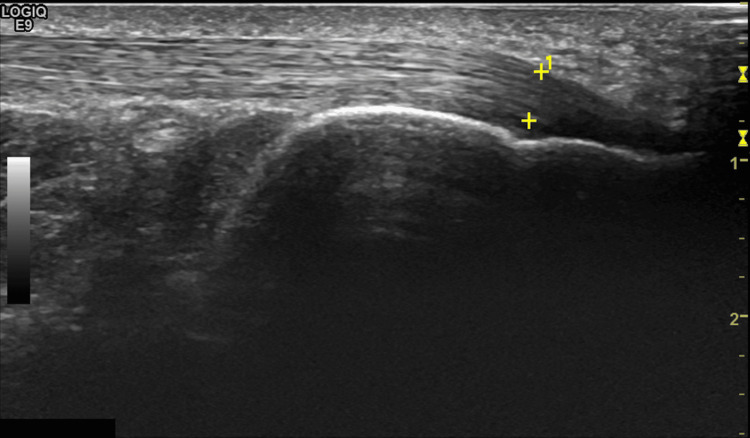
Ultrasound imaging: longitudinal image of Achilles tendon showing a normal fibrillar pattern

Out of the 25 operated patients, nine conceded to visit the hospital for a more detailed long-term follow-up with ultrasonography (four patients in Group A and five patients in Group B). These patients were examined at an average of two years postoperatively by an experienced musculoskeletal radiologist using a LOGIQ E9 diagnostic ultrasound with a 6-15 MHZ linear transducer, comparing the operated side with the other unaffected side (Figure 2).

The quality of the tendon was evaluated in terms of thickness, fibrillar architecture, mobility, presence of calcifications, neovascularization, and enthesopathy. A scoring system between 0 and 11 was used, with 11 being the worst score (Table 1).

**Table 1:** Ultrasound scoring system

B-mode evaluation
Thickness: Antero-posterior tendon thickness was measured on the same longitudinal median scan at two different levels: at the insertion of the tendon’s deeper margin into the calcaneal bone and 4 cm more proximal.
0 - Normal thickness (<6 mm)
1 - Minor thickening (6-8 mm)
2 - Moderate thickening (8-10 mm)
3 - Severe thickening (>10 mm)
*Note*: Double measurements at the insertion and 4 cm cephalad
Structure fibrillar disruption
0 - Normal structure (homogenous echogenicity)
1 - Minor structural changes (discrete hypo-echogenic areas)
2 - Moderate structural changes (some well-defined hypo-echogenic areas)
3 - Severe structural changes (extended hypo-echogenic areas)
Calcifications enthesopathy
0 - No calcifications
1 - Calcifications
Mobility evaluation
0 - Normal gliding
1 - Limited gliding
Color Doppler evaluation - neovascularization (visible high blood flow)
0 - No neovascularization
1 - Mild neovascularization (a few solitary blood vessels)
2 - Moderate neovascularization (moderate quantity, mostly transversal blood vessels)
3 - Severe neovascularization (several, mostly horizontal, blood vessels spread in the whole depth of the tendon)
Maximum score: 11

Statistical analysis

Due to the small sample size, a non-parametric Wilcoxon test (SPSS v24, IBM Corp., Armonk, NY) was used to compare VISA-A and ultrasonography scores between the two groups. The level of significance was set to a = 0.05.

## Results

The mean VISA-A score for the early functional rehabilitation protocol group was 90.1 (SD = 9.87), while it was 83.8 (SD = 17.06, p = 0.624) for the control group. The mean ultrasound score for the early functional rehabilitation protocol group was 7.75 (SD = 1.71), while it was 7.60 (SD = 3.05, p = 0.414) for the control group (Tables 2, 3, 4).

**Table 2:** VISA-A and ultrasonography scores There was no statistical difference between the two groups for both VISA-A scores (mean Group A = 90.1 [SD = 9.87] vs mean Group B = 83.8 [SD = 17.06, p = 0.624]) and ultrasound scores (mean Group = 7.75 [SD = 1.71] vs mean Group B = 7.60 [SD = 3.05, p = 0.414]). VISA-A: Victorian Institute of Sports Assessment-Achilles; US: Ultrasound.

Treatment Group	Patient ID	VISA-A Score	US Score
A (Early functional)	1	94	10
	2	93	7
	3	94	6
	4	81	8
	5	100	
	6	94	
	7	97	
	8	96	
	9	85	
	10	67	
B (Control)	11	92	11
	12	99	4
	13	94	5
	14	80	8
	15	93	10
	16	94	
	17	71	
	18	81	
	19	87	
	20	94	
	21	76	
	22	86	
	23	29	
	24	87	
	25	94	

**Table 3:** Statistical analysis (VISA-A scores) Mean VISA-A scores: Group A = 90.1 (SD = 9.87); Group B (control) = 83.8 (SD = 17.06); P-value = 0.624. VISA-A: Victorian Institute of Sports Assessment-Achilles; Asymp. Sig. (2-tailed): Asymptotic significance (two-tailed).

Descriptive Statistics	N	Mean	Std. Deviation	Minimum	Maximum
Early functional VISA	10	90.1000	9.87083	67.00	100.00
Classic VISA	15	83.8000	17.05956	29.00	99.00
Wilcoxon Signed Ranks Test (Ranks)		N	Mean Rank	Sum of Ranks	
Classic VISA - early functional VISA	Negative ranks	5a	4.30	21.50	
	Positive ranks	3b	4.83	14.50	
	Ties	2c			
	Total	10			
Note:	a. Classic VISA < Early functional VISA	b. Classic VISA > Early functional VISA	c. Classic VISA = Early functional VISA		
Test Statistics (a)	Classic VISA - Early functional VISA				
Z	-.491 (b)				
Asymp. Sig. (2-tailed)	.624				
Note:	a. Wilcoxon signed ranks test	b. Based on positive ranks			

**Table 4:** Statistical analysis (ultrasonography scores) Mean ultrasound scores: Group A = 7.75 (SD = 1.71); Group B (control) = 7.60 (SD = 3.05); P-value = 0.414. VISA-A: Victorian Institute of Sports Assessment-Achilles; US: Ultrasound; Asymp. Sig. (2-tailed): Asymptotic significance (two-tailed).

Descriptive Statistics	N	Mean	Std. Deviation	Minimum	Maximum
Early functional US	4	7.7500	1.70783	6.00	10.00
Classic US	5	7.6000	3.04959	4.00	11.00
Wilcoxon Signed Ranks Test (Ranks)		N	Mean Rank	Sum of Ranks	
Classic US - early functional US	Negative ranks	2a	2.25	4.50	
	Positive ranks	1b	1.50	1.50	
	Ties	1c			
	Total	4			
Note:	a. Classic US < Early functional US	b. Classic US > Early functional US	c. Classic US = Early functional US		
Test Statistics (a)	Classic US - Early Functional US				
Z	-.816 (b)				
Asymp. Sig. (2-tailed)	.414				
Note:	a. Wilcoxon signed ranks test	b. Based on positive ranks.			

As far as the patients are concerned, only one patient was female and belonged to the control group. The mean age of the patients was 39 years, and all of them were operated in the first three days after their injury. None of these patients was a professional athlete.

There were no intra- or early postoperative complications, such as infections or re-ruptures, which is one of the main risks of early rehabilitation protocol, and all patients were satisfied with the results of their operation. Stiffness after the operation was noted in the first three months in one patient from the control group (B), but no other complications were reported. As far as postoperative pain is concerned, no patient reported significant pain. All patients have returned to their preoperative status and daily activities in six months.

Overall, VISA-A and ultrasound scores of the early functional rehabilitation protocol group were better at the last follow-up than that of the control group, although this difference was not significant in this small sample study. The final outcome for the operated patients was satisfactory, and no major differences were reported between the two groups, allowing early return to daily activities for the patients of the early rehabilitation protocol group.

## Discussion

Achilles tendon rupture treatment is still debated in the literature. However, early mobilization reduces the risk of adhesions and postoperative stiffness, minimizes the need for physiotherapy, and increases patients' satisfaction rates. Immediate ambulation also may protect against deep vein thrombosis.

In a randomized study, Cetti et al. concluded that operated patients have a higher rate of return to daily activities and sports, better range of motion, and fewer re-ruptures compared to non-operatively treated patients [20]. This statement is supported by a meta-analysis of randomized control trials by Soroceanu et al., which demonstrates less risk of re-ruptures in the operative treatment group [21]. On the other hand, surgery is related to a higher risk of complications such as deep vein thrombosis [24], infection, postoperative stiffness, and neurovascular injury [25]. In our study, only one of the patients belonging to the classic rehabilitation group reported stiffness that needed prolonged physiotherapy up to three months postoperatively. No other complications were reported.

The results of our study are in accordance with the current literature. The American Academy of Orthopaedic Surgeons published guidelines proposing weight bearing from the first postoperative day [26]. Many authors have reported good outcomes with accelerated rehabilitation, following surgical repair of Achilles rupture. Brumann et al. reviewed the literature on rehabilitation after Achilles repair, reporting better outcomes after early weight bearing and mobilization, and proposed an accelerated rehabilitation protocol that allows for full weight bearing from day one [27]. Similarly, Kim et al. proposed weight bearing after the second week postoperatively [28]. Another study by Bevoni et al. reported good results, in terms of Achilles scores (American Orthopaedic Foot and Ankle Society [AOFAS], Leppilahti), range of movement, and isokinetic dynamometry on patients who followed an accelerated rehabilitation protocol [14]. Huang et al. in a systematic review with meta-analysis concluded that postoperative care should include combined early weight bearing with early ankle joint range of motion exercises [29]. Finally, Zhao et al., in a systematic review, reported that early functional rehabilitation was superior to cast immobilization after Achilles tendon repair [30].

Our study adds to the existing knowledge in favor of an accelerated rehabilitation protocol after Achilles tendon repair. Early return to normal activities after Achilles tendon repair is of great importance, especially for young, active persons. The greatest advantage of our accelerated rehabilitation protocol was that the patients were allowed to perform their exercises unsupervised at home, thus reducing the need for visits to the hospital, and this approach did not have a negative effect on results. However, the findings of our study are limited both by its small sample size and the retrospective design.

## Conclusions

The results of this small sample study showed that an early unsupervised functional rehabilitation protocol allows early mobilization with better functional outcomes after surgical repair of Achilles tendon tears compared to more conservative, supervised physiotherapy protocols. Thus, complications related to prolonged immobilization are avoided, along with the visits to the hospital or physiotherapist's office.
